# Modulation of gut microbiota dysbioses in type 2 diabetic patients by macrobiotic Ma-Pi 2 diet

**DOI:** 10.1017/S0007114516001045

**Published:** 2016-05-06

**Authors:** Marco Candela, Elena Biagi, Matteo Soverini, Clarissa Consolandi, Sara Quercia, Marco Severgnini, Clelia Peano, Silvia Turroni, Simone Rampelli, Paolo Pozzilli, Mario Pianesi, Francesco Fallucca, Patrizia Brigidi

**Affiliations:** 1 Department of Pharmacy and Biotechnology, University of Bologna, 40126 Bologna, Italy; 2 Institute of Biomedical Technologies, Italian National Research Council, 20090 Segrate, Milan, Italy; 3 Department of Endocrinology and Diabetes, University Campus Bio-Medico, 00128 Rome, Italy; 4 International Study Center for Environment, Agriculture, Food, Health and Economics, 62029 Tolentino, Italy; 5 In Unam Sapientiam, La Sapienza University, 00161 Rome, Italy

**Keywords:** Type 2 diabetes, Gut microbiota, Dysbiosis, Fibre-rich diets, Macrobiotic diets

## Abstract

The gut microbiota exerts a role in type 2 diabetes (T2D), and deviations from a mutualistic ecosystem layout are considered a key environmental factor contributing to the disease. Thus, the possibility of improving metabolic control in T2D by correcting gut microbiome dysbioses through diet has been evaluated. Here, we explore the potential of two different energy-restricted dietary approaches – the fibre-rich macrobiotic Ma-Pi 2 diet or a control diet recommended by Italian professional societies for T2D treatment – to correct gut microbiota dysbioses in T2D patients. In a previous 21-d open-label MADIAB trial, fifty-six overweight T2D patients were randomised to the Ma-Pi 2 or the control diet. For the present study, stools were collected before and after intervention from a subset of forty MADIAB participants, allowing us to characterise the gut microbiota by 16S rRNA sequencing and imputed metagenomics. To highlight microbiota dysbioses in T2D, the gut microbiota of thirteen normal-weight healthy controls were characterised. According to our findings, both diets were effective in modulating gut microbiome dysbioses in T2D, resulting in an increase of the ecosystem diversity and supporting the recovery of a balanced community of health-promoting SCFA producers, such as *Faecalibacterium*, *Roseburia*, *Lachnospira*, *Bacteroides* and *Akkermansia*. The Ma-Pi 2 diet, but not the control diet, was also effective in counteracting the increase of possible pro-inflammatory groups, such as *Collinsella* and *Streptococcus*, in the gut ecosystem, showing the potential to reverse pro-inflammatory dysbioses in T2D, and possibly explaining the greater efficacy in improving the metabolic control.

Type 2 diabetes (T2D) is markedly increasing its prevalence in Westernised countries^(^ ^1^ ^)^, and it represents a challenging problem for national healthcare systems^(^ ^2^ ^)^. Recent insights provided evidence of an altered gut microbiota (GM) in T2D subjects, suggesting a possible role for gut micro-organisms in the disease onset^(^ ^3^ ^–^ ^7^ ^)^.

Intestinal micro-organisms, and their metabolic products, have been shown to exert relevant functions in regulating host metabolic pathways^(^ ^8^ ^)^. Indeed, GM components can modulate different factors contributing to the host metabolic phenotype, such as intestinal gluconeogenesis, insulin sensitivity, lipid accumulation and glucose control^(^ ^9^ ^,^ ^10^ ^)^. Although a mutualistic GM composition is crucial to support the host energy homeostasis, certain GM dysbioses can result in profound deregulations of the host metabolism, supporting the onset and consolidation of metabolic diseases, such as T2D^(^ ^11^ ^,^ ^12^ ^)^. Moreover, a pro-inflammatory layout of the gut microbial ecosystem has been suggested to be the basis of chronic inflammatory processes observed in T2D, and the new concept of metabolic infection has been proposed^(^ ^13^ ^,^ ^14^ ^)^. As a result of an increased gut permeability, endotoxins from pro-inflammatory GM components can penetrate the epithelial barrier and aggravate metabolic inflammation and insulin resistance in T2D^(^ ^15^ ^)^. Thus, the GM has the potential to exert a multifactorial role in T2D, and deviations from a health-promoting GM composition could represent a key determinant contributing to the disease onset^(^ ^11^ ^,^ ^16^ ^–^ ^18^ ^)^. As diet has been recognised as a potent modulator of the composition and metabolism of the human GM^(^ ^19^ ^)^, the possibility to improve metabolic control in T2D by developing selective diets that are able to correct the GM dysbioses has been considered^(^ ^11^ ^,^ ^16^ ^,^ ^20^ ^)^.

Very recently, the Ma-Pi 2 macrobiotic diet has been reported to be more effective than a control (CTR) diet, which is based on the dietary guidelines recommended by professional societies in Italy, for the improvement of metabolic control in T2D patients^(^ ^21^ ^)^. Conceived by Mario Pianesi, the Ma-Pi 2 diet is a fibre-rich macrobiotic diet with a prebiotic potential, which is in line with the dietary recommendations of the Academy of Nutrition and Dietetics^(^ ^22^ ^)^. Specifically, the Ma-Pi 2 diet is enriched in complex carbohydrates, legumes, fermented products, sea salt and green tea, and it excludes fat and protein from animal source and added sugars. In a 21-d controlled open-label trial (MADIAB trial), fifty-six overweight T2D patients were randomised (1:1 ratio) to the Ma-Pi 2 macrobiotic diet or the CTR diet. At the end of this short-term nutritional intervention, the Ma-Pi 2 macrobiotic diet proved to be more effective in reducing fasting and postprandial blood glucose, glycated Hb (HbA1c), serum cholesterol, homeostasis model assessment of insulin resistance (HOMA-IR), BMI and waist and hip circumferences compared with the CTR diet^(^ ^21^ ^)^. According to the authors, the greater effect of the high-fibre Ma-Pi 2 diet on several metabolic parameters of T2D patients was probably because of, at least in part, an inherent capability of favouring the recovery of a mutualistic GM layout. To verify this hypothesis, in this study, we specifically compared the efficacy of the Ma-Pi 2 and the CTR diet in modulating GM dysbioses in a subset of forty overweight T2D patients participating in the MADIAB trial, for whom stools were successfully collected before and after intervention. To this aim, stools were analysed for the microbiota composition by next-generation sequencing (NGS) of the 16S rRNA gene and imputed metagenomics. To provide a picture of the baseline GM dysbioses in the enrolled T2D patients, their microbiota profiles were compared with those of thirteen normal-weight healthy controls. Our findings suggest that the Ma-Pi 2 diet has the potential to reverse compositional and functional GM dysbioses in T2D, favouring the recovery of a mutualistic configuration capable of supporting the host energy homeostasis.

## Methods

### Study design

The design of the MADIAB trial is described in Soare *et al*.^(^ ^21^ ^)^. Briefly, it was designed as a 21-d controlled open-label trial, in which the participants were assigned (1:1) to the Ma-Pi 2 macrobiotic diet or a CTR diet based on the dietary guidelines for T2D recommended by professional societies in Italy. The trial was conducted in accordance with the Declaration of Helsinki and the Good Clinical Practice guidelines, and the study was approved by the Institutional Review Board of University Campus Bio-Medico (trial registration number ISRCTN10467793; http://www.isrctn.com/ISRCTN10467793). Written informed consent was obtained from all subjects/patients. The Department of Endocrinology and Diabetes of the University Campus Bio-Medico in Rome (Italy) recruited overweight or obese (BMI 27–45 kg/m^2^) subjects, aged 40–77 years and affected by T2D (Table 1). Associated metabolic syndrome was evaluated according to the National Cholesterol Education Program Adult Treatment Panel III criteria, although it was not an inclusion criterion. Inclusion criteria were as follows: T2D diagnosed at least 1 year before the start of the trial, treated exclusively with dietary intervention, oral hypoglycaemic drugs or both for 6 months before study entry. Exclusion criteria were as follows: the use of insulin either at present or at any time in the 2 year before the study, current use of corticosteroid therapy or any other drug that can interfere with carbohydrate metabolism, alcohol abuse and pregnancy. Subjects who already followed a macrobiotic diet were excluded from the study. Participants’ eating habits concerning the period antecedent the study start were assessed using qualitative and quantitative questionnaires (online Supplementary Table S1). Eligible T2D subjects were divided into two groups according to the diet randomly assigned (twenty-eight participants were randomised to the Ma-Pi 2 diet and twenty-eight to the CTR diet), and they were accommodated in two different hotels, which were located close to each other. Randomisation was stratified by BMI at baseline and by sex.

**Table 1:** Anthropometric characteristics of type 2 diabetes (T2D) patients, measured at T0 and T1 (after 21-d Ma-Pi 2 or control diet (CTR) intervention), and normal-weight healthy controls (Mean values and standard deviations)

No.	Arm	Age (years)	Sex	Weight T0 (kg)	Mean weight T0 (kg)	sd	Weight T1 (kg)	Mean weight T1 (kg)	sd	BMI T0 (kg/m^2^)	Mean BMI T0 (kg/m^2^)	sd	BMI T1 (kg/m^2^)	Mean BMI T1 (kg/m^2^)	sd
1	Ma-Pi 2	50	F	99·1	88·4	17·5	93·5	82·8	16·5	41·4	34·3	6·5	39·1	32·2	6·1
2	Ma-Pi 2	72	M	108·6			103·2			34·1			32·4		
3	Ma-Pi 2	77	F	72·8			68·7			35			33		
4	Ma-Pi 2	67	M	88			82·5			30			28·2		
5	Ma-Pi 2	72	F	104			96·4			50·7			47		
6	Ma-Pi 2	62	M	72·4			68·6			24·3			23		
7	Ma-Pi 2	76	M	130			121·7			45·5			42·6		
8	Ma-Pi 2	63	F	108·4			100·5			43·2			40·1		
9	Ma-Pi 2	59	M	95·7			89·1			35·4			33		
10	Ma-Pi 2	75	F	73·9			70			33·2			31·5		
11	Ma-Pi 2	75	F	60·1			55·2			28·9			26·5		
12	Ma-Pi 2	73	M	88·9			82·9			30·7			28·7		
13	Ma-Pi 2	70	F	75·8			71·5			31·3			29·5		
14	Ma-Pi 2	71	F	81·1			77·2			30·9			29·5		
15	Ma-Pi 2	62	M	74·7			69·2			29·6			27·4		
16	Ma-Pi 2	57	F	116·9			109·3			36·8			34·4		
17	Ma-Pi 2	60	M	81			75·2			27·7			25·6		
18	Ma-Pi 2	76	M	77·6			72			29·9			27·7		
19	Ma-Pi 2	61	F	75·6			69·9			31·3			29		
20	Ma-Pi 2	72	F	77·7			73·3			31·1			29·3		
21	Ma-Pi 2	66	F	93·4			88·8			40			38		
1	CTR	60	F	122·2	88·2	15·5	119	85·5	14·9	50·7	32·1	6·3	49·3	31·1	6·1
2	CTR	56	M	103·1			98·6			36·5			34·9		
3	CTR	51	M	88·3			85·9			33·7			32·8		
4	CTR	57	F	88			85·9			33·1			32·3		
5	CTR	72	F	55·9			55·3			22			22		
6	CTR	73	F	77			75·2			30·8			30·1		
7	CTR	74	M	89·3			86·8			27·3			26·5		
8	CTR	68	M	80·7			78·8			24·9			24·4		
9	CTR	74	M	72·8			69·6			28·8			27·5		
10	CTR	72	M	78·5			75·1			28·2			27		
11	CTR	53	M	105·4			100·2			32·1			30·6		
12	CTR	69	M	85·9			83·3			28·1			27·2		
13	CTR	74	F	80·7			77·8			33·6			32·3		
14	CTR	67	M	104·5			100·6			36·2			34·9		
15	CTR	62	M	80·3			78·3			29·5			28·8		
16	CTR	69	M	104·2			101·2			34·8			33·8		
17	CTR	63	F	85·7			83·5			31·9			31·1		
18	CTR	64	F	72·4			69·3			27·6			26·4		
19	CTR	65	F	101·8			99·8			40·7			39·9		
1	IT1	38	M	78·2	65·6	10·7	N/A	N/A	N/A	22·9	22·0	1·8	N/A	N/A	N/A
2	IT2	34	F	62·4			N/A			20			N/A		
3	IT3	29	F	60·9			N/A			22·4			N/A		
4	IT4	27	M	87·2			N/A			22·9			N/A		
5	IT5	30	M	72·3			N/A			23·2			N/A		
6	IT6	32	F	58·4			N/A			22·7			N/A		
7	IT9	39	F	54·3			N/A			20·3			N/A		
8	IT10	40	F	53·1			N/A			18·3			N/A		
9	IT11	40	F	67·6			N/A			24·8			N/A		
10	IT12	30	F	57·5			N/A			22			N/A		
11	IT14	21	F	59·3			N/A			21·3			N/A		
12	IT15	29	M	80·4			N/A			24·6			N/A		
13	IT16	32	F	61·3			N/A			21·2			N/A		

F, female; M, male; IT, Italian healthy controls.

Stratified random sampling was used to ensure that the groups contained similar numbers of patients with BMI≥35·0 kg/m^2^ (the median in the cohort of eligible patients) and similar number of male patients. The menus were designed as a 2-d diet repeated cyclically along the whole duration of the study. Both diets were energy intake restricted to 7949 kJ (1900 kcal) for men and 7113 kJ (1700 kcal) for women (online Supplementary Table S2). In particular, the CTR diet derived energy from 40–60 % carbohydrate, 10–20 % protein, 30 % fat and ≥20 g/4184 kJ (1000 kcal) fibre. It was adapted to the Mediterranean culinary style. Vegetables, fruit, cereal, fish and white meat typical of the Mediterranean style were used; alcohol and sucrose consumption was forbidden. Diet meal plans and recipes are described in the Additional File 1 of the study by Soare *et al*.^(^ ^21^ ^)^. Briefly, daily diet administration was organised in three meals (breakfast, lunch and dinner) and two snacks, 2·5 h after breakfast and lunch. Every participant was informed that a leftover ≥5 % of the total food intake meant the dismissal from the trial. Participants were asked to keep their exercise habits unvaried during the intervention period, and their physical activity was registered using a pedometer (Tri-axial activity monitor, XL-18/XL-18 CN-AND; A&D Medical). Primary outcomes were the change in fasting blood glucose (FBG) and postprandial blood glucose (PPBG) levels from the baseline (T0) to the 21st day of treatment (T1). Secondary outcomes included changes from baseline in plasma concentration of HbA1c, total cholesterol, LDL-cholesterol, HDL-cholesterol and the LDL:HDL ratio, C-reactive protein (CRP), TNF-*α* and IL-6, as well as insulin resistance, body weight, BMI, waist and hip circumference and GM composition. For GM analysis, participants were asked to provide a faecal sample before (T0) and after the 21-d nutritional intervention (T1). Stool samples were successfully collected from a subset of forty T2D patients participating in the MADIAB trial aged 50–77 years (mean age 66 years): twenty-one assigned to the Ma-Pi 2 diet and nineteen to the CTR diet. The gut microbiome study was conducted in this subset of forty MADIAB participants.

In addition, thirteen healthy controls, aged 21–40 years (mean age 32 years) and with 18·3–24·6 kg/m^2^ BMI, were enrolled for the study (Table 1). They were asked to provide a faecal sample, and their eating habits were assessed using qualitative and quantitative questionnaires as well (online Supplementary Table S1). All samples were immediately frozen at −20°C, and then transferred within 1 week to −80°C and stored there until processing.

### 16S *ribosomal* DNA sequencing and processing

Total microbial DNA was extracted from faeces using the DNeasy Blood & Tissue Kit (Qiagen) by introducing three FastPrep (MP Biomedicals) bead-beating 1-min steps at 5·5 movements/s, with 5-min incubation in ice between treatments^(^ ^23^ ^)^. DNA recovery was evaluated using the NanoDrop ND-1000 spectrophotometer (NanoDrop Technologies) and 2200 TapeStation instrument (Agilent Technologies). For each sample, the V3–V4 region of the 16S rRNA gene was PCR-amplified in 25-μl volumes containing 12·5 ng of microbial DNA, 2× KAPA HiFi HotStart ReadyMix (Kapa Biosystems) and 200 nmol/l of S-D-Bact-0341-b-S-17/S-D-Bact-0785-a-A-21 primers^(^ ^24^ ^)^ carrying Illumina overhang adapter sequences (Bio-Fab Research). Thermal cycle consisted of an initial denaturation at 95°C for 3 min, twenty-five cycles of denaturation at 95°C for 30 s, annealing at 55°C for 30 s, extension at 72°C for 30 s and a final extension step at 72°C for 5 min. Amplicons of about 460 bp were purified with a magnetic bead-based clean-up system (Agencourt AMPure XP; Beckman Coulter) and sequenced on Illumina MiSeq platform using a 2×300 bp paired end protocol, according to the manufacturer’s instructions (Illumina). Briefly, indexed libraries were prepared by limited-cycle PCR using Nextera technology and further cleaned up with AMPure XP magnetic beads (Beckman Coulter). Libraries were pooled at equimolar concentrations, denatured and diluted to 6 pmol/l before loading onto the MiSeq flow cell. Amplicon sequences were deposited in the MG-RAST database (http://metagenomics.anl.gov/linkin.cgi?project=17675).

### Bioinformatics and statistics

Raw sequences were processed using a pipeline combining PANDAseq (paired-end assembler for Illumina sequences)^(^ ^25^ ^)^ and QIIME (Quantitative Insights Into Microbial Ecology)^(^ ^26^ ^)^. High-quality reads were binned into operational taxonomic units (OTU) at a 0·97 similarity threshold using UCLUST^(^ ^27^ ^)^. Taxonomy was assigned using the RDP (Ribosomal Database Project) classifier against Greengenes database (May 2013 release). Chimera filtering was performed by discarding all singleton OTU. *α* Rarefaction was analysed by using the Faith’s phylogenetic diversity, Chao1, observed species and Shannon index metrics. *β* Diversity was estimated by computing weighted and unweighted UniFrac distances. Weighted UniFrac distances were used for principal coordinates analysis (PCoA) and plotted by the rgl and vegan packages of R. Data separation in the PCoA was tested using a permutation test with pseudo *F* ratios (function adonis in the vegan package). Heat-map analysis was performed using the R ggplot2 package.

Metagenome imputation of Greengenes-picked OTU was performed using PICRUSt (Phylogenetic Investigation of Communities by Reconstruction of Unobserved States)^(^ ^28^ ^)^ with default settings. The KEGG (Kyoto Encyclopedia of Genes and Genomes) Orthology (KO) database^(^ ^29^ ^)^ was used for functional annotation. Procrustes superimposition was conducted on the normalised KO gene data set and phylogenetic compositional data using vegan and rgl.

The correlation between age and GM diversity was computed by Kendall *τ* correlation test. To identify which feature in each diet contributed to the modulation of single bacterial groups, a graphical representation of phylogenetic data and dietary metadata was obtained using GraPhlAn^(^ ^30^ ^)^.

All statistical analyses were performed in R, version 3.1.3. Significant differences were assessed by Wilcoxon’s signed rank-sum test. When appropriate, a paired test was used. Where necessary, *P* values were corrected for multiple comparisons using the Benjamini–Hochberg method. *P*<0·05 was considered statistically significant.

Statistical analysis of the primary and other secondary outcomes from the subset of forty MADIAB participants who provided stool samples was carried out as reported by Soare *et al*.^(^ ^21^ ^)^. Briefly, quantitative variables were summarised using percentiles, and the comparison between time points and dietary groups was performed using the Wilcoxon’s signed rank-sum test.

## Results

### Comparison of the gut microbiota compositional structure between overweight type 2 diabetes patients and healthy controls

The phylogenetic structure of the GM ecosystem of forty overweight T2D patients before (T0) and after (T1) nutritional intervention, and thirteen normal-weight healthy adults, was characterised by NGS of the V3–V4 region of the 16S ribosomal DNA (rDNA) (online Supplementary Fig. S1). A total of 3 198 509 high-quality reads were obtained, with a mean of 30 277 (sd 4850) reads per sample. Reads were clustered in 30 874 OTU at 97 % identity. Rarefaction curves obtained with Shannon, Chao1, PD (phylogenetic diversity) whole tree and observed species phylogenetic metrics approximated the saturation level after 3300 reads (online Supplementary Fig. S1).

**Fig. 1:**
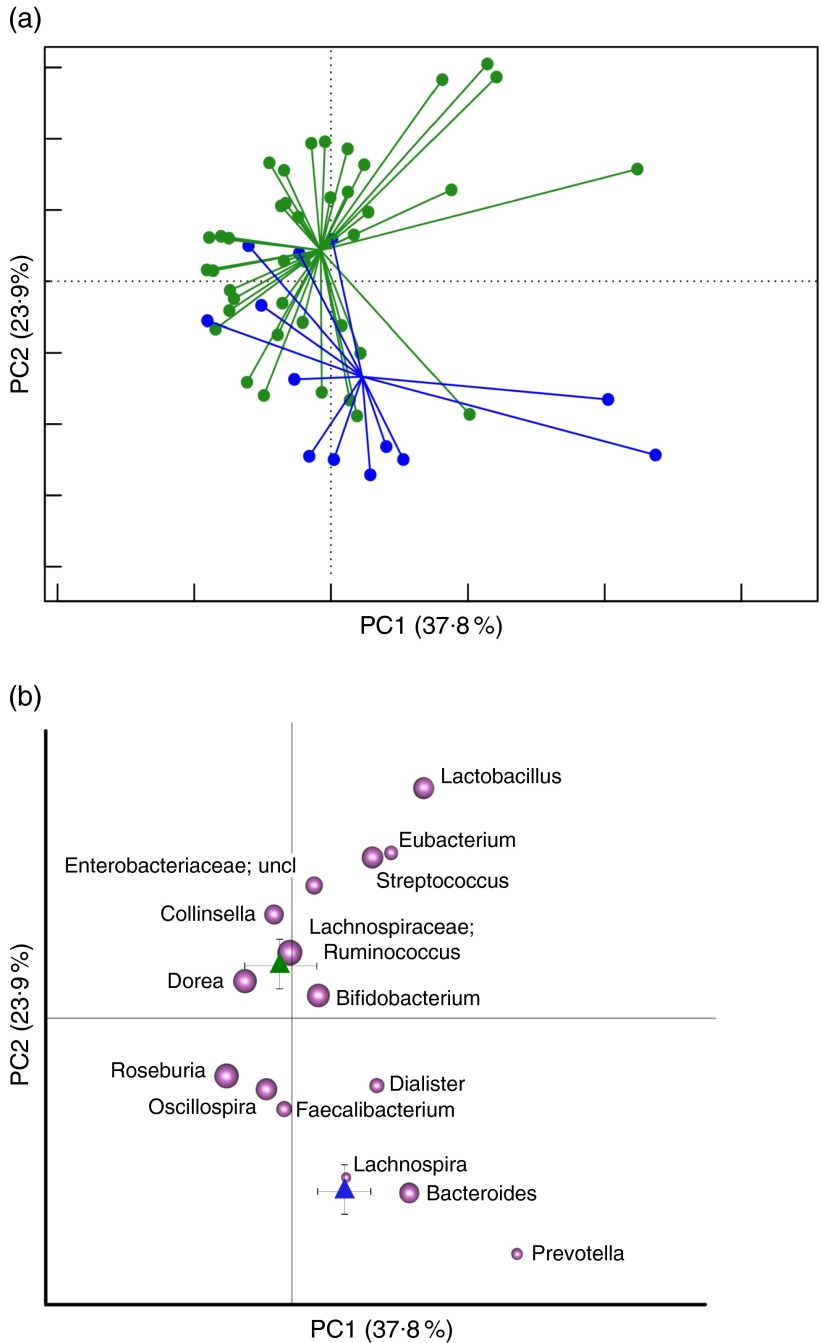
Comparison of the gut microbiota compositional structure between overweight type 2 diabetes (T2D) patients at baseline and healthy controls. (a) Principal coordinates analysis (PCoA) based on weighted UniFrac distances shows separation between forty overweight T2D patients at T0 and thirteen normal-weight healthy controls. 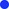, Healthy controls; 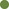, T2D patients. *P*<0·001; permutation test with pseudo *F* ratios. (b) Superimposition of microbial genera on the PCoA plot in order to identify the genera involved in this separation. Sphere width is proportional to the mean relative abundance of the genus across all samples. The two components explain 37·8 and 23·9 % of the variance, respectively. 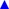, 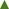, Centroids for each group with indication of standard errors on each coordinate axis; uncl, unclassified.

To characterise GM dysbioses in T2D at the enrolment, the compositional structure of the GM at T0 was compared with that of healthy controls. In the online Supplementary Table S1, we provide the dietary behaviours during the 6 months before the trial for all T2D patients, as well as the dietary habits of the healthy controls. T2D patients were characterised by a significant reduction of the GM Shannon diversity index (*P*<0·05, Wilcoxon’s signed rank-sum test). Even if it cannot be excluded that the age differences between T2D patients (mean age 66 years) and healthy controls (mean age 32 years) contribute, at least in part, to the observed differences in GM diversity, we failed to detect any significant correlation between age and microbiome diversity in our data set (online Supplementary Fig. S2). The PCoA of the weighted UniFrac distances resulted in a significant segregation between the two groups (Fig. 1(a)) (*P*<0·001, permutation test with pseudo *F* ratios), confirming the presence of compositional differences in the GM structure of T2D patients and healthy controls. To identify the microbial genera responsible for this separation, the biplot of the average bacterial coordinates weighted by the corresponding bacterial abundance per sample was superimposed on the PCoA plot (Fig. 1(b)), as previously carried out by Lozupone *et al*.^(^ ^31^ ^)^. The GM genera that clustered with T2D patients and healthy controls and the corresponding relative abundance values are reported in Table 2. According to our biplot analysis, T2D patients showed a higher load of several potentially pro-inflammatory GM components, such as Enterobacteriaceae, *Collinsella* ^(^ ^32^ ^)^ and *Streptococcus*. In particular, Enterobacteriaceae, *Collinsella* and *Streptococcus* were, respectively, 8·5, 3·5 and 4·1 times more in T2D patients than in healthy controls. T2D patients also showed a significantly higher abundance of *Lactobacillus*, with an increase of about 150 times, and Lachnospiraceae:*Ruminococcus*, which was 1·1 times more than in healthy controls. Conversely, T2D patients were slightly depleted in several health-promoting SCFA producers, such as *Bacteroides* (0·7 times less), *Prevotella* (0·8 times less), *Lachnospira* (0·7 times less), *Roseburia* (0·2 times less) and *Faecalibacterium* (0·4 times less). Taken together, our data demonstrate the presence of dysbioses in the GM of T2D patients, characterised by an overall reduction of mutualistic GM components in favour of a corresponding increase in potential pro-inflammatory groups.

**Table 2:** Relative genus abundance in forty overweight type 2 diabetes (T2D) patients and thirteen normal-weight healthy controls (Percentages and standard deviations)*

Genera	Rel. Ab. (T2D, %)	sd	Rel. Ab. (healthy, %)	sd	*P*
*Bifidobacterium*	3·32	0·05	7·23	0·05	0·007
*Collinsella*	2·67	0·02	0·60	0·01	0·01
*Bacteroides*	3·05	0·04	10·8	0·09	0·002
*Streptococcus*	3·62	0·07	0·73	0·01	0·09
*Dorea*	2·06	0·01	1·56	0·01	0·1
*Lachnospira*	0·47	0·01	1·55	0·01	0·0006
*Roseburia*	5·38	0·07	6·98	0·05	0·07
Lachnospiraceae:*Ruminococcus*	4·98	0·05	2·33	0·02	0·01
*Faecalibacterium*	4·95	0·04	7·85	0·05	0·02
*Oscillospira*	1·18	0·01	1·71	0·01	0·04
Unclassified Enterobacteriaceae	1·79	0·03	0·19	0·01	0·2
*Prevotella*	0·42	0·01	1·97	0·04	0·1
*Eubacterium*	1·18	0·02	0·26	0·01	0·08
*Dialister*	1·08	0·02	0·95	0·02	0·8
*Lactobacillus*	2·98	0·08	0·02	0·01	0·0002

Rel.Ab., relative abundance.

* Relative *P* values calculated by applying the Wilcoxon’s signed rank-sum test are also reported

### Structural modulation of the gut microbiota of type 2 diabetes patients after nutritional intervention

T2D patients participating in the MADIAB trial were randomised (1:1 ratio) to follow the Ma-Pi 2 or the CTR diet. Primary and secondary outcomes for the fifty-one patients who completed the trial were provided by Soare *et al*.^(^ ^21^ ^)^. As stool samples for gut microbiome characterisation were successfully collected before and after intervention from twenty-one patients assigned to the Ma-Pi 2 diet and nineteen patients assigned to the CTR diet, primary and secondary outcomes were re-analysed in this subset of forty MADIAB participants. The obtained data confirmed a significant reduction of FBG and PPBG in both diet groups, which was significantly higher for patients following the Ma-Pi 2 diet compared with those following the CTR diet. With regard to secondary outcomes, the Ma-Pi 2 diet group showed a significantly higher reduction in HOMA-IR, total cholesterol, LDL-cholesterol and LDL:HDL ratio compared with the CTR group (Table 3). Furthermore, although both diets were effective in reducing the plasma TNF-*α* levels, only the Ma-Pi 2 dietary intervention resulted in a significant reduction in plasma levels of CRP and IL-6 (Table 4).

**Table 3:** Primary and secondary outcome comparison between Ma-Pi 2 and control (CTR) diet at T0 and T1 (Medians and 1st–3rd quartile ranges)

			Ma-Pi 2 diet (*n* 21)					CTR diet (*n* 19)				
	T0		T1			T0		T1				
	Median	1st–3rd quartile	Median	1st–3rd quartile	*P* *	Median	1st–3rd quartile	Median	1st–3rd quartile	*P* †	*P* ‡	*P* §
Fasting blood glucose (mg/l)	1260	1110; 1530	950	850; 1000	0·007	1380	1125; 1645	1080	1025; 1150	0·004	0·7	0·0002
Postprandial blood glucose (mg/l)	1270	1090; 1790	1000	940; 1060	0·009	1470	1360; 2130	1275	1117; 1537	0·009	0·03	0·0002
HbA1c (%)	6·5	6·10; 7·7	6·1	5·8; 7·0	0·2	6·9	6·4; 7·5	6·8	6·2; 7·1	0·4	0·7	0·3
HOMA-IR	3·3	1·4; 4·2	1·0	0·6; 2·1	0·0004	3·2	0·9; 4·6	1·4	1·1; 2·2	0·08	0·8	0·1
Total cholesterol (mg/l)	1780	1550; 2290	1230	1030; 1390	0·0002	1830	1520; 2140	1560	1375; 1895	0·1	0·9	0·01
LDL-cholesterol (mg/l)	1020	870; 1340	630	430; 870	0·0003	1050	880; 1270	850	670; 1045	0·07	0·9	0·03
HDL-cholesterol (mg/l)	490	390; 520	440	380; 510	0·5	500	440; 570	470	405; 570	0·7	0·4	0·3
LDL:HDL ratio	2·4	2·0; 3·0	1·5	0·9; 1·9	0·004	2·0	1·9; 2·8	1·8	1·4; 2·3	0·2	0·2	0·2
Weight (kg)	81·1	75·6; 99·1	77·2	70·0; 93·5	0·2	85·9	79·4; 102·5	83·3	75·2; 92·7	0·2	0·8	0·7
BMI (kg/m^2^)	31·3	30·0; 36·8	29·5	28·2; 34·4	0·2	30·8	27·8; 34·2	30·1	26·9; 33·3	0·4	0·1	0·4
Waist circumference (cm)	108·1	100·3; 114·7	103·4	95·8; 11·7	0·2	105·5	101·8; 112·8	102·9	100·1; 108·0	0·4	0·6	0·9
Hip circumference (cm)	109·5	103·3; 123·8	105·3	101·8; 120·0	0·4	109·1	100·6; 117·5	107·5	99·5; 115·1	0·6	0·5	0·8

HbA1c, glycated Hb; HOMA-IR, homeostasis model assessment of insulin resistance.

* *P* values indicated for T0 and T1 for Ma-Pi 2 diet.

† *P* values indicated for T0 and T1 for CTR diet.

‡ *P* values indicated for Ma-Pi 2 and CTR diet at T0.

§ *P* values indicated for Ma-Pi 2 and CTR diet at T1.

**Table 4:** Inflammatory marker comparison between Ma-Pi 2 and control (CTR) diet at T0 and T1 (Medians and 1st–3rd quartile ranges)

			Ma-Pi 2 diet (*n* 21)					CTR diet (*n* 19)				
	T0		T1			T0		T1				
	Median	1st–3rd quartile	Median	1st–3rd quartile	*P* *	Median	1st–3rd quartile	Median	1st–3rd quartile	*P* value†	*P* value‡	*P* value§
CRP (mg/l)	3·2	1·2; 11·8	1·0	0·3; 2·1	0·004	2·7	1·4; 5·9	1·6	1·1; 4·9	0·4	0·8	0·03
TNF-*α* (pg/ml)	23·3	0·0; 35·7	0·0	0·0; 2·1	0·001	26·2	11·2; 38·9	4·0	0·5; 8·4	0·002	0·4	0·01
IL-6 (pg/ml)	4·3	0·8; 6·4	2·9	2·4; 5·1	0·006	3·3	0·1; 5·5	3·5	2·1; 5·8	0·4	0·2	0·6

CRP, C-reactive protein.

* *P* values indicated for T0 and T1 for Ma-Pi 2 diet.

† *P* values indicated for T0 and T1 for CTR diet.

‡ *P* values indicated for Ma-Pi 2 and CTR diet at T0.

§ *P* values indicated for Ma-Pi 2 and CTR diet at T1.

**Fig. 2:**
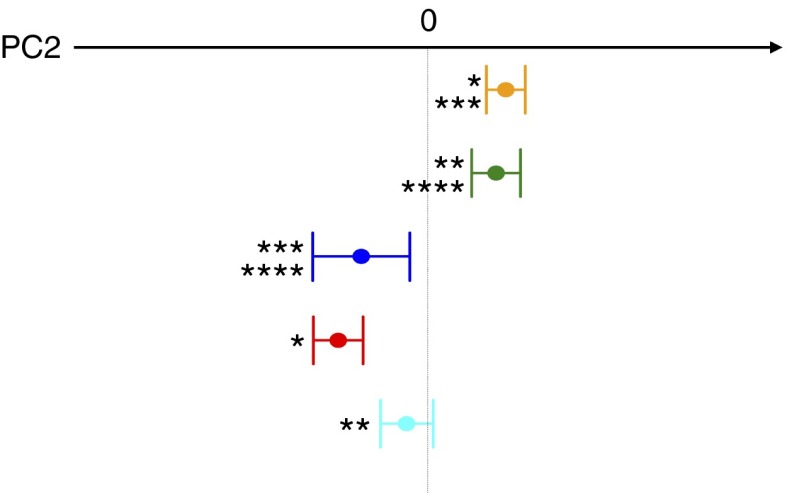
Variation of the weighted UniFrac PC2 coordinates between the study groups. 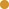, Ma-Pi 2 diet group at T0; 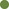, control (CTR) diet group at T0; 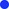, healthy controls; 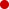, Ma-Pi 2 diet group at T1; 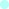, CTR diet group at T1. For each group, average (±sem, error bar) PC2 coordinates are shown. The significance of the differences between the PC2 coordinates of the groups is indicated as follows: * *P*<0·001 (Ma-Pi 2 diet group at T0 *v.* T1), ** *P*=0·01 (CTR diet group at T0 *v.* T1), *** *P*=0·01 (Ma-Pi 2 diet group at T0 *v.* healthy controls), **** *P*=0·03 (CTR diet group at T0 *v.* healthy controls); Wilcoxon’s signed rank-sum test.

We then explored the impact of dietary interventions on the GM ecosystem of T2D patients. To visualise the total variation in the data set, a PCoA analysis of the weighted UniFrac distances between the Ma-Pi 2 and CTR groups at T0 and T1 and the healthy controls was performed (online Supplementary Fig. S3). The PC2 axis – which accounts for 24 % of the total variability – was positively associated with T2D (*P*<0·05, Wilcoxon’s signed rank-sum test). Thus, the efficacy of dietary interventions in redressing T2D-associated GM-dysbioses was assessed by comparing the variation of the average (±sem) PC2 coordinates for each group of samples (Fig. 2). Although at T0 both Ma-Pi 2 and CTR diet groups showed significantly higher PC2 coordinates compared with healthy controls (*P*=0·01 and *P*=0·03, respectively), at T1 no significant difference between both diet groups and healthy controls was observed. These data support the potential of both dietary interventions to redress T2D-associated GM dysbioses, favouring the recovery of a mutualistic ecosystem layout.

**Fig. 3:**
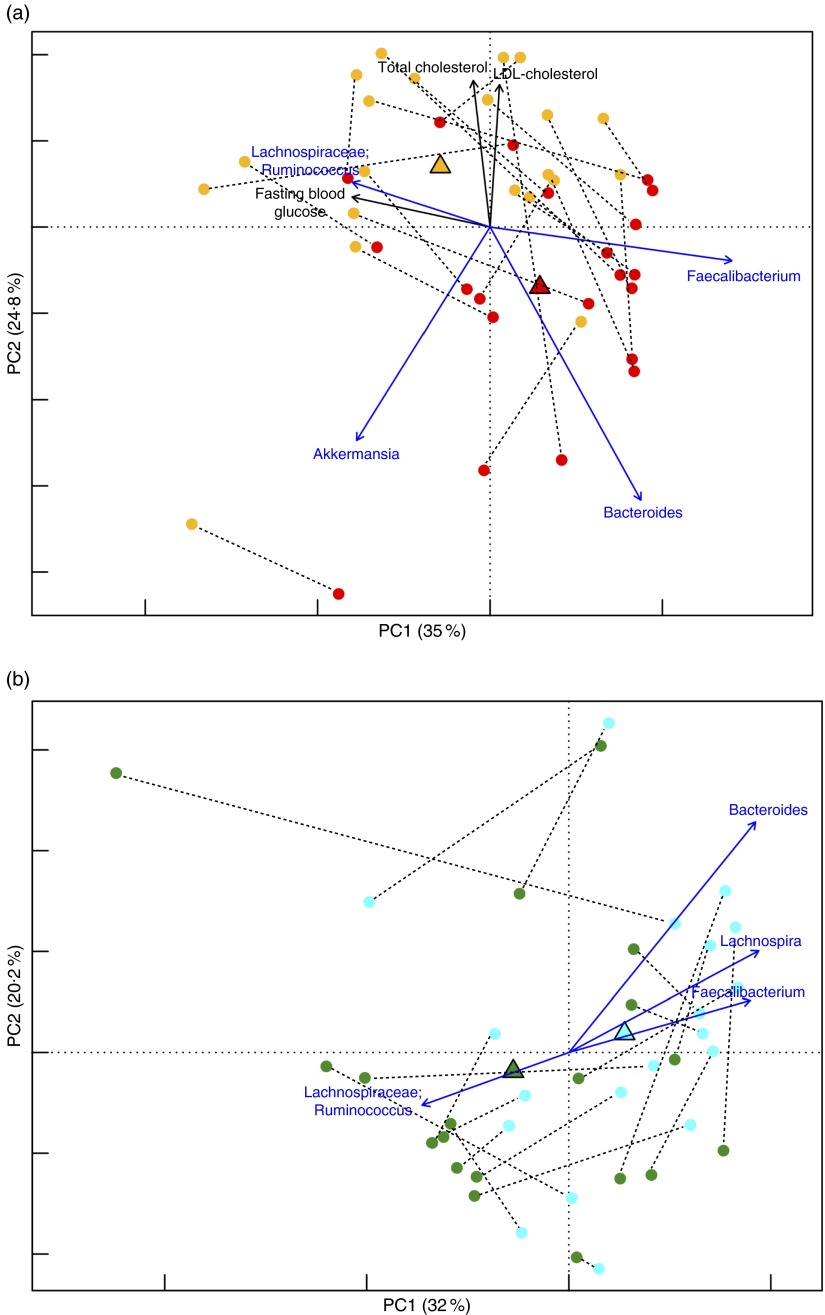
Comparison of the gut microbiota compositional structure of type 2 diabetes (T2D) patients before and after the nutritional interventions. (a) Principal coordinates analysis (PCoA) based on weighted UniFrac distances for T2D subjects following the Ma-Pi 2 diet (*n* 21) shows separation between T0 (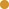) and T1 (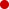). The two components explain 35·0 and 24·8 % of the variance, respectively. *P*=0·01; permutation test with pseudo *F* ratios. (b) PCoA based on weighted UniFrac distances for T2D subjects following the control (CTR) diet (*n* 19). 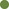, T0; 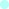, T1. The two components explain 32·0 and 20·2 % of the variance, respectively. *P*=0·04; permutation test with pseudo *F* ratios. Lines connect T0 and T1 samples from the same patient. 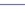, 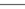 responding bacterial genera and biochemical parameters, respectively; 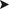, direction of significant correlations; 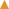, 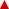, 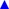, 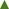, centroids for each time point.

The specificity of each dietary intervention in correcting the GM dysbioses in T2D patients was evaluated by comparing the compositional structure of the GM ecosystem before (T0) and after (T1) the nutritional intervention, as reported in the two PCoA plots of weighted UniFrac distances, which also included the responding bacterial genera and biochemical parameters (Fig. 3). A significant segregation between the GM profiles at T0 and T1 was obtained for both interventions (*P*=0·01 and *P*=0·04 for Ma-Pi 2 and CTR diet, respectively; permutation test with pseudo *F* ratios), indicating that both diets were effective in modulating the GM of T2D patients. Although both diets tended to increase the GM Shannon diversity (*P*=0·09 and *P*=0·06 for Ma-Pi 2 and CTR diet, respectively; Wilcoxon’s signed rank-sum test), no significant difference in within-group diversity between time points was observed (Fig. 3). Interestingly, in the case of the Ma-Pi 2 diet, we detected significant associations between changes in GM components and changes in biochemical parameters (Fig. 3(a)). In particular, *Faecalibacterium*, which increased at T1, showed an inverse association with FBG. Analogously, *Bacteroides* and *Akkermansia*, both positively responding to the Ma-Pi 2 diet, showed an inverse trend with respect to total and LDL-cholesterol. Conversely, Lachnospiraceae:*Ruminococcus*, decreasing after the Ma-Pi 2 diet, was positively associated with FBG.

**Fig. 4:**
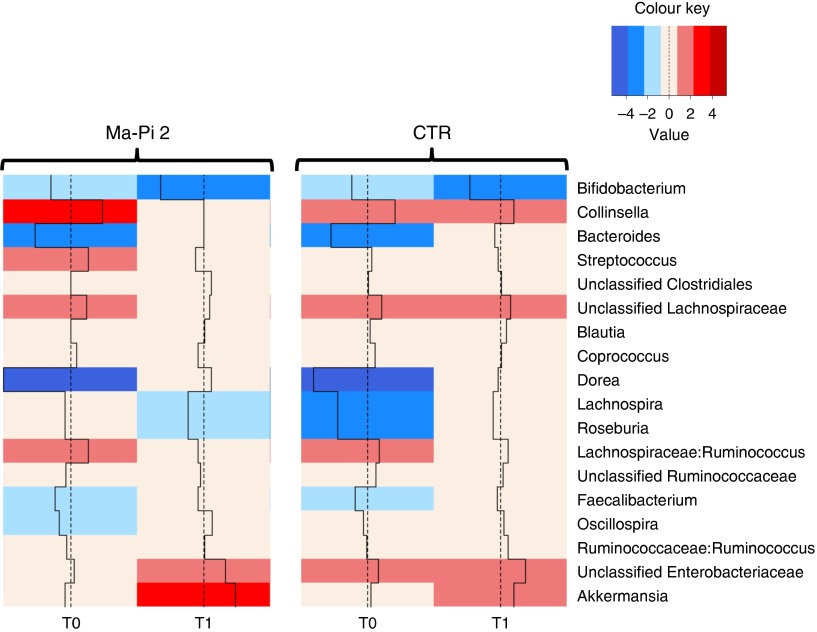
Impact of dietary interventions on the taxonomic structure of the core microbiota in type 2 diabetes (T2D) patients. Heat maps were calculated for both Ma-Pi 2 and control (CTR) diet, based on the log-ratio of the median genus abundance in T2D patients (T0 and T1 samples) and healthy controls. The colour code and segment line reveal the deviation in terms of fold change from the median profile of healthy subjects (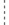). T2D patients following the Ma-Pi 2 diet (*n* 21); T2D patients following the CTR diet (*n* 19); healthy controls (*n* 13).

To dissect the specific GM compositional changes in response to the dietary regimen, we focused our analysis on the core GM community, defined as the sum of the microbial genera present at a relative abundance >1 % in at least 30 % of the subjects. Selected genera accounted together for >80 % of the total ecosystem. To estimate the degree of dysbiosis of the core GM in T2D, the ratio of the median genus abundance in T2D patients and healthy controls was calculated. The heat map of the log-ratios at T0 and T1 for the Ma-Pi 2 and the CTR diet is shown in Fig. 4. According to our findings, both diets were effective in counteracting the decrease of *Bacteroides*, *Dorea* and *Faecalibacterium* in T2D patients, as demonstrated by the recovery of median abundance values similar to those of healthy controls. Moreover, both Ma-Pi 2 and CTR diet resulted in an increase of *Akkermansia* above the abundance values characteristic of healthy controls. On the other hand, both diets supported the reduction of Lachnospiraceae:*Ruminococcus* down to the median abundance values shown by healthy controls. Diet-specific effects on the GM composition of T2D patients regarded only a few genera under-represented in T2D patients compared with healthy controls: *Lachnospira* and *Roseburia*, for which only the CTR diet favoured the recovery of health-like median abundance values, and *Oscillospira*, for which the recovery of health-like values occurred only in the Ma-Pi 2 diet group. Conversely, the genera *Collinsella* and *Streptococcus*, and unclassified Lachnospiraceae, which were enriched in T2D patients compared with healthy controls, decreased down to health-like abundances only in patients who underwent the Ma-Pi 2 diet intervention.

**Fig. 5:**
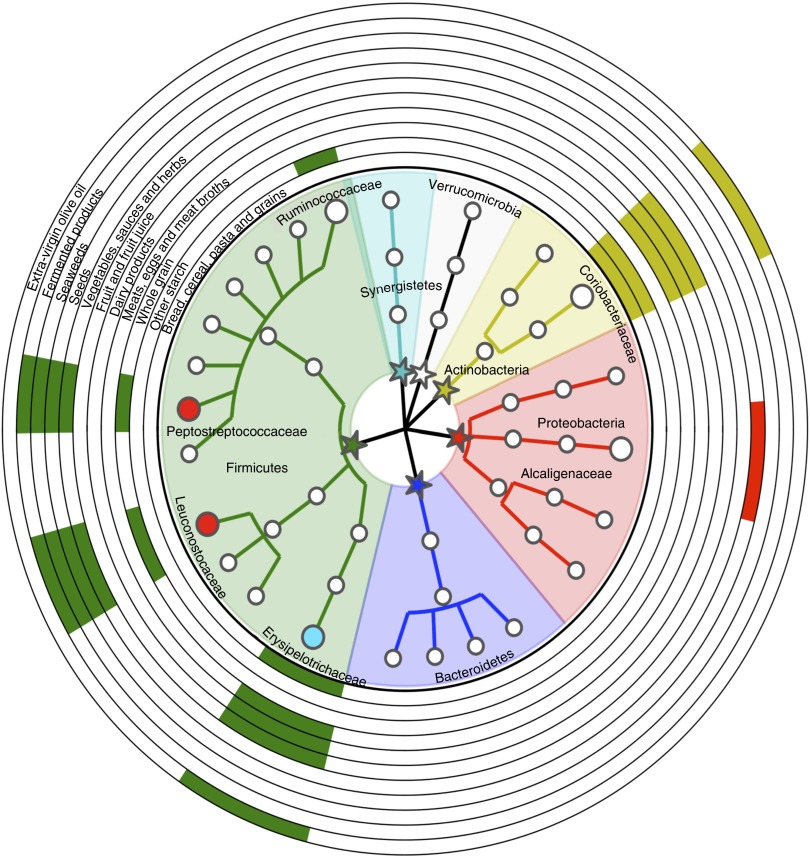
Impact of dietary components on the gut microbiota taxonomic structure. Cladogram obtained with the GraPhlAn tool, showing the family-level gut microbiota profile of T1 samples from both intervention groups with a phylum-based colour code (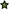, Firmicutes; 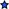, Bacteroidetes; 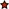, Proteobacteria; 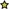, Actinobacteria; 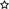, Verrucomicrobia; 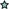, Synergistetes). Families with relative abundance of at least 0·5 % in at least two samples are plotted. Larger circles identify bacterial families having a positive correlation with at least one dietary component; the names of these families are reported. Filled circles identify bacterial families that showed significantly higher abundance in T1 samples of the Ma-Pi 2 diet group (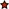) or the CTR diet group (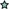). Bacterial family-food component correlations are indicated by filled boxes in the external rings of the plot, referring to the list of dietary components. T2D patients following the Ma-Pi 2 diet (*n* 21); T2D patients following the CTR diet (*n* 19).

To highlight specific dietary features affecting the GM composition, a comparison between the microbiota profiles at T1 of the two groups was performed, taking into account also the dietary features of the two different interventions (Fig. 5). T1 samples from the Ma-Pi 2 diet group showed significantly higher abundance of Peptostreptococcaceae (*P*=0·02; Wilcoxon’s signed rank-sum test) and Leuconostocaceae (*P*=0·0002), which were also positively correlated with the ingested amount of dietary components that were represented in a higher percentage in Ma-Pi 2 compared with the CTR diet (whole grain 28·1 *v.* 8·5 %, vegetables, sauces and herbs 64·5 *v.* 32·0 %), or only present in the Ma-Pi 2 diet (seeds, seaweeds and fermented products) (see also online Supplementary Table S2). On the contrary, bacteria from the family Erysipelotrichaceae showed significantly higher abundance in T1 samples from the CTR diet group (*P*=0·008), and were positively correlated with the ingested amount of dietary components present only in CTR diet (bread, cereal, pasta and grains, meats, eggs and meat broths, dairy products, fruit and fruit juice and extra-virgin olive oil). The same positive correlations were obtained for Coriobacteriaceae, even if the abundance of this bacterial family was not significantly higher in the CTR diet group T1 samples. This bacterial group also showed a positive correlation with the food category ‘other starch’ (containing legumes and potatoes), which was more represented in the CTR diet than in the Ma-Pi 2 diet (5·5 *v.* 3·0 %). This food category was also correlated to the Ruminococcaceae family.

### Impact of nutritional intervention on the functional configuration of the gut microbiome of type 2 diabetes patients

To gain insight into the GM functions, gut metagenomes structures were inferred from the respective phylogenetic profiles using PICRUSt^(^ ^28^ ^)^, as previously performed by David *et al*.^(^ ^19^ ^)^ and Morgan *et al*.^(^ ^33^ ^)^. A total of 329 KEGG pathways were generated, of these 249 showed a significantly different abundance between healthy controls and T2D patients, and thirty-four showed a significant variation in response to the Ma-Pi 2 diet, whereas no KEGG pathway responded significantly to the CTR diet. The Procrustes analysis of the 16S rDNA sequences and imputed KO gene data set co-illustrates the data, supporting the significant association between taxonomic and inferred functional profiles of the gut microbiome across our study cohort (*P*=0·001, Protest; online Supplementary Fig. S4). The PCoA of the imputed functional GM profiles showing segregation among the Ma-Pi 2 and CTR groups at T0 and T1 and healthy controls is provided as the online Supplementary Fig. S5.

**Fig. 6:**
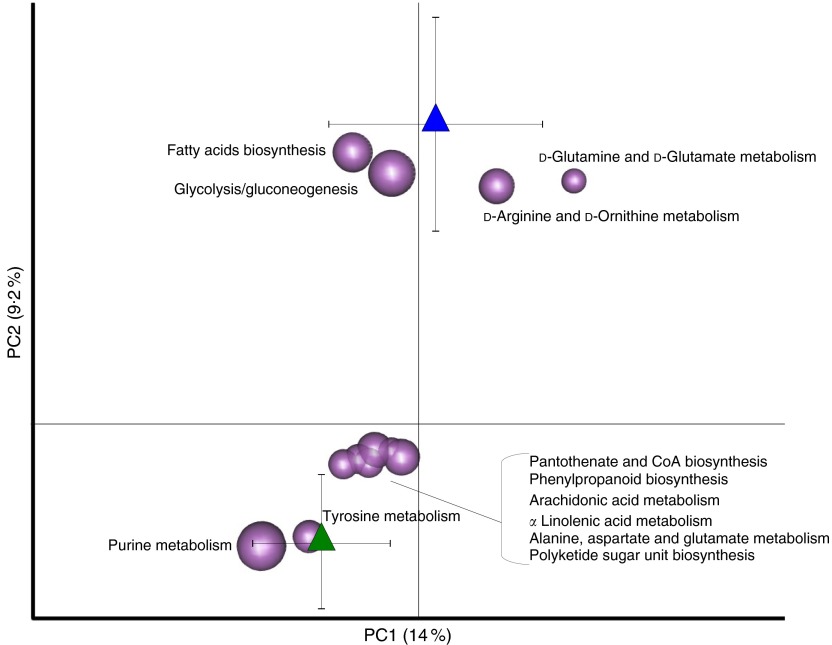
Functional dysbioses of the gut microbiome in type 2 diabetes (T2D) patients. Metabolic pathways were superimposed on the principal component analysis plot based on Euclidean distances, and the pathways responsible for the separation are shown. 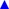, Healthy controls (*n* 13); 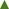, T2D patients (*n* 40). Sphere width is proportional to the mean relative abundance of the function across all samples. The two components explain 14·0 and 9·2 % of the variance, respectively. *P*=0·03; permutation test with pseudo *F* ratios. 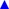, 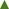, Centroids for each group with indication of standard errors on each coordinate axis.

The Euclidean principal component analysis (PCA) of the KO level gene abundances showed separate clustering between the gut microbiome structure of T2D patients at T0 and healthy controls (*P*=0·03, permutation test with pseudo *F* ratios). To identify functions responsible for this separation, the average coordinates of the metabolic pathways in all samples, weighted by gene count per sample, were obtained and the resulting biplot of pathway distribution was superimposed on the PCA plot (Fig. 6). The pathways clustering with T2D patients and healthy controls are listed in the online Supplementary Table S3. In the context of this functional separation, T2D patients showed a general perturbation in microbiome pathways involved in the metabolism of amino acids, lipids and secondary metabolites. In particular, T2D patients were depleted in genes involved in the metabolism of d-arginine and d-ornithine, as well as of d-glutamine and d-glutamate, while being enriched in pathways involved in tyrosine metabolism and alanine, aspartate and glutamate metabolism. This functional layout may lead to a reduced production of arginine – as a result of both the reduction of pathways directly involved in arginine biosynthesis and the lower amount of glutamate available for arginine biosynthesis – and to a corresponding increase in alanine levels. Although arginine has been reported as insulinogenic^(^ ^34^ ^)^, an increased alanine production has been connected to augmented energy intake, cholesterol level and BMI^(^ ^35^ ^)^. Furthermore, an increased tyrosine metabolism in T2D patients could result in higher levels of the 4-cresol metabolite, which has been associated with diverse inflammatory conditions in the gut^(^ ^36^ ^)^. Finally, compared with healthy controls, the GM of T2D patients showed increased abundance of functions involved in the arachidonic acid metabolism. Interestingly, the bacterial metabolism of this key inflammatory intermediate is a well-recognised immune-escaping strategy for proteobacteria^(^ ^37^ ^)^. A higher level of arachidonic acid metabolism in the GM of T2D patients, together with the increase in the biosynthesis of polyketide sugars – bacterial secondary metabolites with a range of biological functions including immunosuppression^(^ ^38^ ^)^ – might be the result of the hypothesised bloom of pro-inflammatory pathobionts in the gut.

**Fig. 7:**
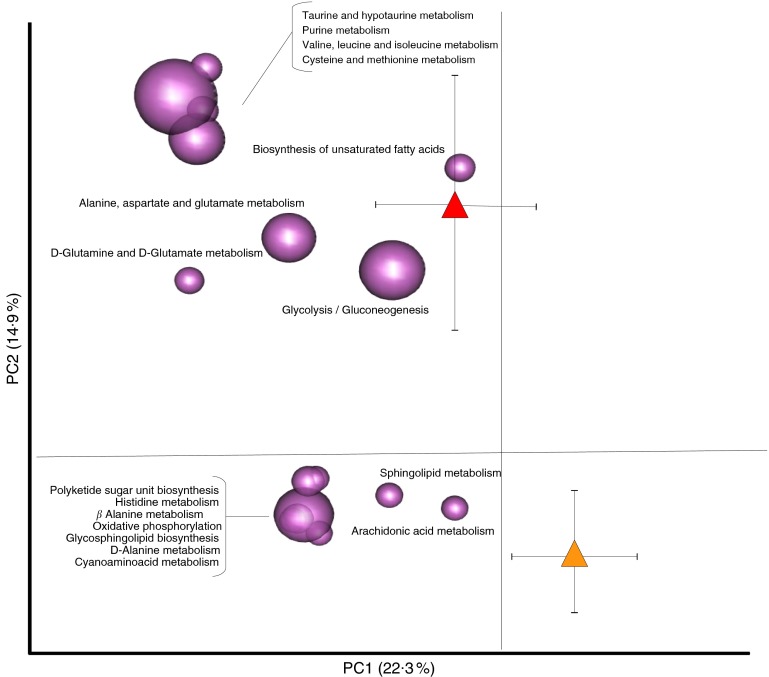
Impact of Ma-Pi 2 dietary intervention on the functional configuration of the gut microbiome in type 2 diabetes (T2D) patients. Metabolic pathways were superimposed on the principal component analysis plot based on Euclidean distances in T2D patients before (T0, 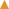) and after (T1, 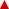) the Ma-Pi 2 diet (*n* 21). The two components explain 22·3 and 14·9 % of the variance, respectively. *P*=0·007; permutation test with pseudo *F* ratios. 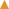, 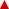, Centroids for each group with indication of standard errors on each coordinate axis.

With the aim to explore the impact of nutritional interventions on the functional configuration of the gut microbiome in T2D patients, the Euclidean PCA of KO level gene abundances at T0 and T1 for both dietary groups was carried out. Differently from the CTR diet, which resulted in no significant functional change in the predicted gut metagenome (*P*=0·4, permutation test with pseudo *F* ratios), the Ma-Pi 2 diet was effective in modulating the GM metagenome in T2D patients, as demonstrated by significant sample clustering according to the time point (*P*=0·007). As previously described, the corresponding biplot of metabolic pathway distribution was superimposed on the PCA plot (Fig. 7). The pathways clustering with the Ma-Pi 2 diet time points are listed in online Supplementary Table S3. According to our findings, the Ma-Pi 2 diet effectively counteracted the functional dysbioses in the GM of T2D patients, resulting in the decrease of the abundance of several T2D-associated GM functional markers, such as alanine metabolism, arachidonic acid metabolism and polyketide sugar biosynthesis, as well as in the reduction of GM functions related to oxidative phosphorylation and glycosphingolipid biosynthesis. Moreover, the Ma-Pi 2 diet favoured an increase in GM functions involved in d-glutamine and d-glutamate metabolism, previously reported as reduced in T2D patients, and in the biosynthesis of unsaturated fatty acids, which have been shown to improve lipoprotein profile, glycaemic control and antioxidant status in T2D^(^ ^39^ ^,^ ^40^ ^)^. Finally, the Ma-Pi 2 diet resulted in the increase of GM pathways involved in the metabolism of taurine, cysteine, methionine, valine, leucine and isoleucine, potentially providing the host with an additional source of important nutrients and essential amino acids.

## Discussion

In the present study, the GM phylogenetic and functional dysbioses in forty overweight T2D patients participating in the MADIAB trial^(^ ^21^ ^)^ were explored. In agreement with previous findings^(^ ^11^ ^)^, T2D involved a significant reduction of the GM diversity, which is a common feature shared by several disease-associated dysbiotic microbiome configurations, such as those associated with inflammatory bowel disease and age^(^ ^41^ ^,^ ^42^ ^)^. According to our results, the reduction of the GM compositional diversity in T2D corresponded to phylogenetic changes. T2D patients were indeed enriched in *Lactobacillus*, Lachnospiraceae:*Ruminococcus* and in several potential pro-inflammatory GM components, such as Enterobacteriaceae, *Collinsella* and *Streptococcus* ^(^ ^32^ ^,^ ^39^ ^,^ ^43^ ^–^ ^46^ ^)^, whereas they were depleted in important health-promoting SCFA producers, such as members of Lachnospiraceae, *Faecalibacterium*, *Bacteroides* and *Prevotella*. Strengthening these findings, analogous T2D-associated GM structural dysbioses have already been identified in previous studies^(^ ^3^ ^,^ ^6^ ^,^ ^7^ ^,^ ^47^ ^)^. Subsequently, we explored the changes in GM functions matching these compositional perturbations by inferred metagenomics. Our data suggest deregulation in pathways involved in the metabolism of amino acids, lipids and secondary metabolites in the GM of T2D patients, including a reduced abundance of functions for the metabolism of d-arginine and d-ornithine, as well as of d-glutamine and d-glutamate, a corresponding increase in the metabolism of tyrosine, alanine, aspartate and glutamate, and a higher load of functions involved in arachidonic acid metabolism and polyketide sugar biosynthesis.

The observed T2D-related dysbiotic microbial community could exert a multifactorial role in the disease onset, contributing to metabolic and immune deregulation. Indeed, the T2D GM is slightly depleted in fibrolytic health-promoting mutualists, fundamental for providing butyrate and propionate from the degradation of indigestible plant polysaccharides and starch, such as the butyrate-producing *Dorea*, *Lachnospira*, *Roseburia* and *Faecalibacterium*, and the propionate-producing *Bacteroides* and *Prevotella* ^(^ ^48^ ^)^. Even if the biological relevance of this depletion of SCFA producers remains to be determined, it could result in the reduction of bioavailability of these crucial GM metabolites in the gut, with consequences on the host metabolic and immunological homeostasis. For instance, butyrate and propionate are important for host glucose control^(^ ^11^ ^)^, insulin sensitivity regulation, insulin signalling and intestinal gluconeogenesis^(^ ^9^ ^,^ ^49^ ^)^; also, they represent potent immune modulators, being involved in peripheral regulatory T-cell generation^(^ ^50^ ^)^ and in the regulation of pro-inflammatory cytokine production^(^ ^51^ ^)^. In parallel, the observed increase of potential pro-inflammatory micro-organisms in the gut of T2D patients, such as Enterobacteriaceae, *Collinsella* and *Streptococcus* ^(^ ^32^ ^,^ ^39^ ^,^ ^43^ ^–^ ^46^ ^)^, could further contribute to raise the host inflammatory level, supporting the evolution of insulin resistance^(^ ^52^ ^)^. Finally, the functional GM layout of T2D patients we obtained by imputed metagenomics suggests additional mechanisms involved in the GM contribution to the disease, including the reduced production of the insulinogenic arginine^(^ ^34^ ^)^, and the increased production of alanine, a recognised marker of higher energy intake and cholesterol level^(^ ^35^ ^)^, which could contribute to the loss of metabolic control in T2D. Moreover, the higher potential for the production of tyrosine metabolites could further boost pro-inflammatory stimuli in the gut^(^ ^36^ ^)^. Interestingly, the survey of GM in T2D patients allowed us to find traces of functions related to immune escaping, such as pathways involved in the metabolism of arachidonic acid and polyketide sugar biosynthesis, further highlighting the ongoing bloom of pro-inflammatory GM components in T2D^(^ ^39^ ^)^. However, the functional conclusions derived from imputed metagenomics must be taken with caution until experimentally confirmed by shotgun metagenomics.

In the subset of forty MADIAB participants – twenty-one assigned to the Ma-Pi 2 diet and nineteen to the CTR diet – we then explored the efficacy of the nutritional interventions in supporting the recovery of a mutualistic GM configuration in T2D patients. Primary and secondary outcomes were re-analysed for this patient subset included in the gut microbiome study, confirming that the Ma-Pi 2 diet was associated with a greater reduction in FBG and PPBG, total serum cholesterol, CRP and IL-6 in T2D patients. According to gut microbiome data, both Ma-Pi 2 and CTR diet were able to modulate the GM dysbioses in T2D patients, supporting the recovery of a healthy-like compositional structure and resulting in an increased ecosystem diversity, which represents a strategic feature for a healthy GM ecosystem^(^ ^53^ ^)^. In addition, both diets supported the recovery of a balanced health-promoting community of fibrolytic SCFA producers in the gut of T2D patients, by re-increasing the abundance of propionate and butyrate producers (i.e. *Bacteroides*, *Dorea*, *Faecalibacterium*) and consolidating a healthy-like abundance of *Roseburia*, *Lachnospira*, Lachnospiraceae:*Ruminococcus* and *Oscillospira*. Moreover, both dietary interventions resulted in the increase of *Akkermansia*, a human mucus coloniser recently correlated to an improved metabolic profile^(^ ^54^ ^)^, providing the rationale for its use in the prevention or treatment of obesity and associated metabolic disorders. Finally, it is important to point out that only the Ma-Pi 2 diet showed the potential to counteract the rise of putative pro-inflammatory components (i.e. *Collinsella* and *Streptococcus*) in the GM ecosystem of T2D patients. When we sought associations between GM components and biochemical parameters, significant trends were obtained only in the case of the Ma-Pi 2 diet. Indeed, in Ma-Pi 2-treated subjects, the abundance of *Faecalibacterium* was negatively associated with FBG and, analogously, the abundance of *Bacteroides* and *Akkermansia* showed an inverse association with total and LDL-cholesterol. Conversely, Lachnospiraceae:*Ruminococcus* was positively associated with FBG. These findings suggest that these micro-organisms, the first increasing and the latter decreasing in response to the Ma-Pi 2 diet, could represent Ma-Pi 2-responding GM components associated with primary and secondary study outcomes. Supporting our findings, the negative association between *Faecalibacterium* and FBG concentration had already been detected in a study exploring the structural modulation of the GM in T2D subjects treated with a Chinese herbal formula^(^ ^55^ ^)^. Moreover, in a study carried out in mice, *Akkermansia muciniphila* treatment has been demonstrated to reverse high-fat diet-induced metabolic disorders, including fat-mass gain, metabolic endotoxaemia, adipose tissue inflammation and insulin resistance^(^ ^54^ ^)^. On the other hand, Lachnospiraceae:*Ruminococcus* has recently been positively correlated with insulin resistance in obese women^(^ ^56^ ^)^. According to imputed metagenomics, only the Ma-Pi 2 diet resulted in a significant modulation of the functional microbiome layout in T2D patients. In particular, the decrease of several markers of functional GM dysbioses in T2D patients, such as imbalances in alanine metabolism, arachidonic acid metabolism and polyketide sugar biosynthesis, was observed. Moreover, the Ma-Pi 2 diet favoured the reduction of GM functions related to oxidative phosphorylation and glycosphingolipids biosynthesis. Anaerobic respiration provides an ecological advantage for Enterobacteriaceae in an inflamed gut^(^ ^39^ ^)^, whereas glycosphingolipids are powerful bacterial modulators of the host inflammatory response^(^ ^40^ ^)^. Thus, the reduction in abundance of these pathways further suggests the potential of Ma-Pi 2 diet to counteract the ongoing bloom of pro-inflammatory pathobionts in T2D. Finally, the Ma-Pi 2 diet favoured the increase of GM functions involved in the biosynthesis of unsaturated fatty acids and essential amino acids, showing an added potential to promote the recovery of metabolic control and a balanced nutritional status in T2D patients.

In conclusion, both Ma-Pi 2 and CTR diets showed the potential to support the recovery of GM-host mutualism in T2D patients, favouring the restoration of fibrolytic SCFA-producing GM components, thus promoting metabolic control of T2D patients. Differently from the CTR diet, the Ma-Pi 2 diet was effective in counteracting the rise of possible pro-inflammatory micro-organisms in T2D patients. This suggests that the Ma-Pi 2 diet may have the potential to reduce GM-dependent pro-inflammatory stimuli in the gut that, increasing chronic inflammation, can lead to insulin resistance in T2D^(^ ^57^ ^)^. Even if direct causation still needs to be proved, and this conclusion must be taken with adequate caution, this peculiar property shown by the Ma-Pi 2 diet could partly explain the greater improvements in metabolic control following Ma-Pi 2 dietary intervention compared with the CTR diet^(^ ^21^ ^)^.

## Supplementary material

For supplementary material accompanying this paper visit http://dx.doi.org/10.1017/S0007114516001045. click here to view supplementary material
