# Supplementary material for: Modulation of gut microbiota dysbioses in type 2 diabetic patients by macrobiotic Ma-Pi 2 diet
Source: Br J Nutr. 2016 May 6;116(1):80–93. doi: 10.1017/S0007114516001045 (PMC4894062; doi:10.1017/S0007114516001045)
Supplement: Supplementary file 1 [file S0007114516001045sup.zip › S0007114516001045sup001.pdf]

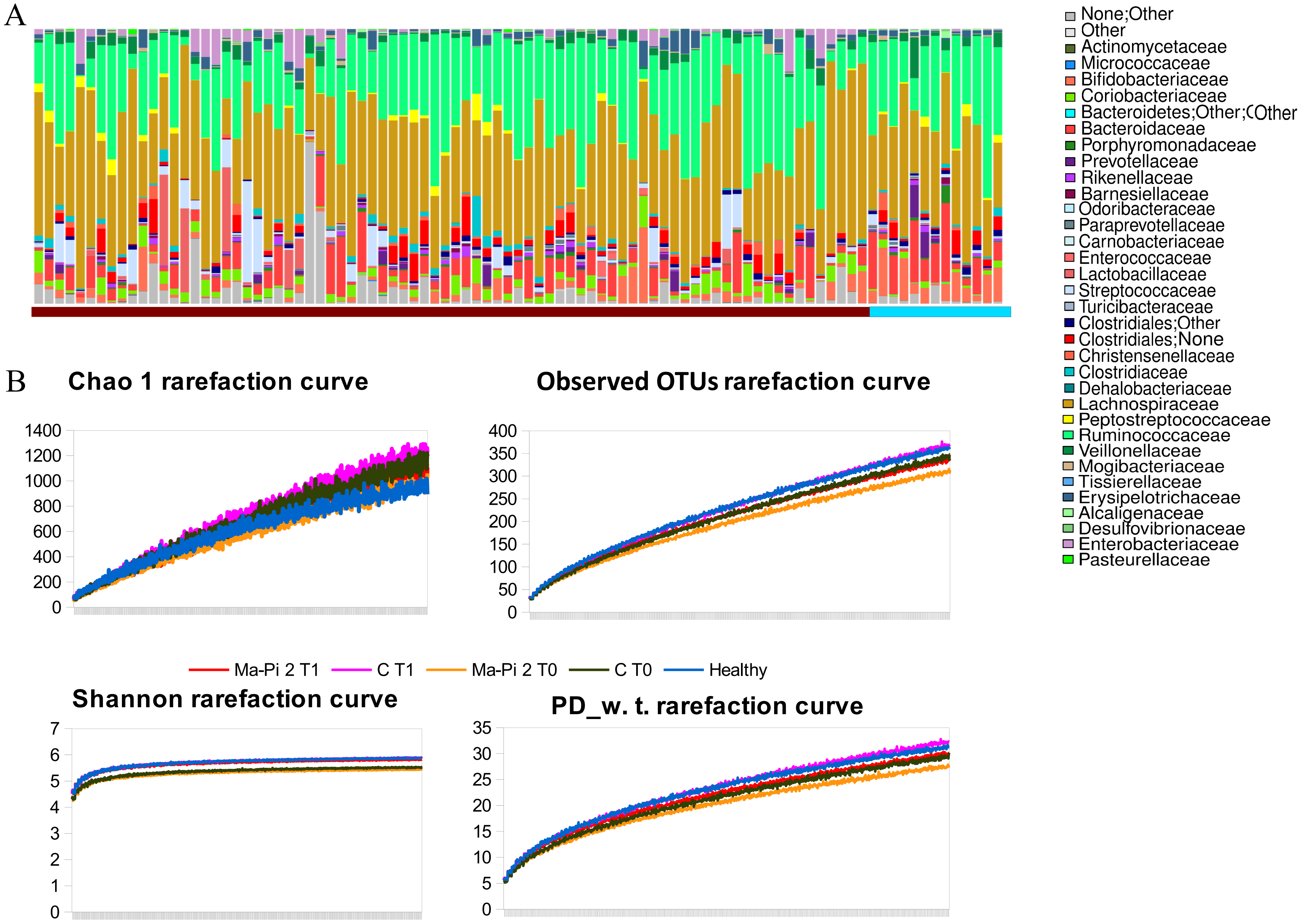

Supplemental Figure 1: Gut microbiota in T2D patients and healthy controls. [A] Phylogenetic structure of the gut microbiota ecosystem of T2D patients (red) and healthy controls (blue); colors were assigned for all families detected. [B] Rarefaction curves with different alpha-diversity metrics: the Chao 1 measure of microbial richness, observed species, the Shannon index of biodiversity and the Faith's phylogenetic diversity (PD_w.t.).
