# Supplementary material for: Modulation of gut microbiota dysbioses in type 2 diabetic patients by macrobiotic Ma-Pi 2 diet
Source: Br J Nutr. 2016 May 6;116(1):80–93. doi: 10.1017/S0007114516001045 (PMC4894062; doi:10.1017/S0007114516001045)
Supplement: Supplementary file 1 [file S0007114516001045sup.zip › S0007114516001045sup002.pdf]

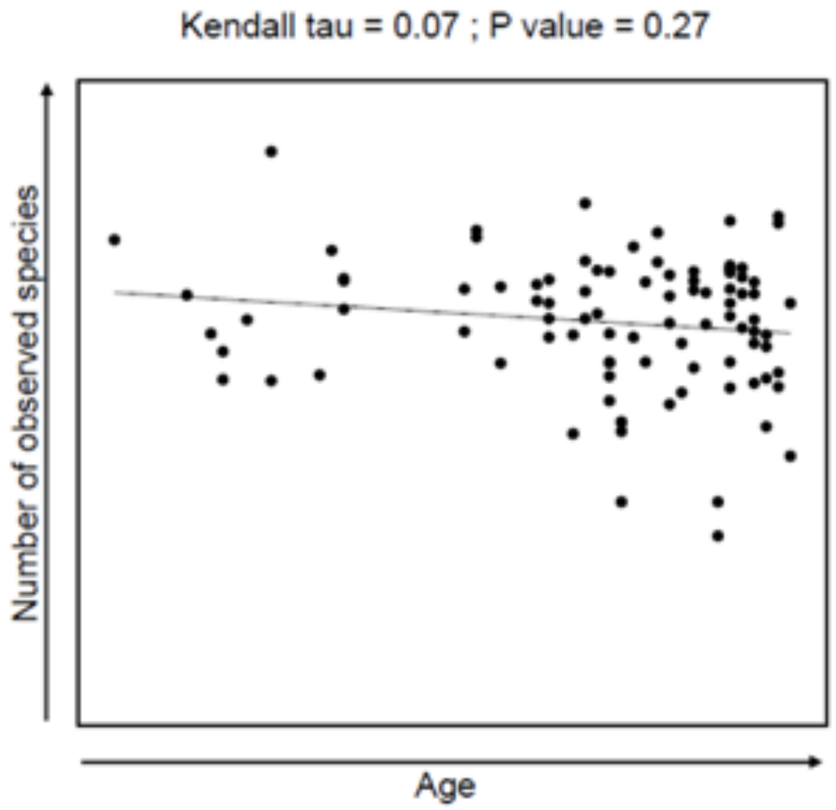

Supplemental Figure 2: Kendall tau correlation between age and gut microbiome diversity in the 40 MADIAB participants and the 13 healthy controls.Excluding a strong age-related variance in microbiome diversity across our study cohorts, no significant correlation between age and biodiversity in our dataset was obtained (Kendall's tau = 0.07; P value = 0.27).
