# Supplementary material for: Modulation of gut microbiota dysbioses in type 2 diabetic patients by macrobiotic Ma-Pi 2 diet
Source: Br J Nutr. 2016 May 6;116(1):80–93. doi: 10.1017/S0007114516001045 (PMC4894062; doi:10.1017/S0007114516001045)
Supplement: Supplementary file 1 [file S0007114516001045sup.zip › S0007114516001045sup003.pdf]

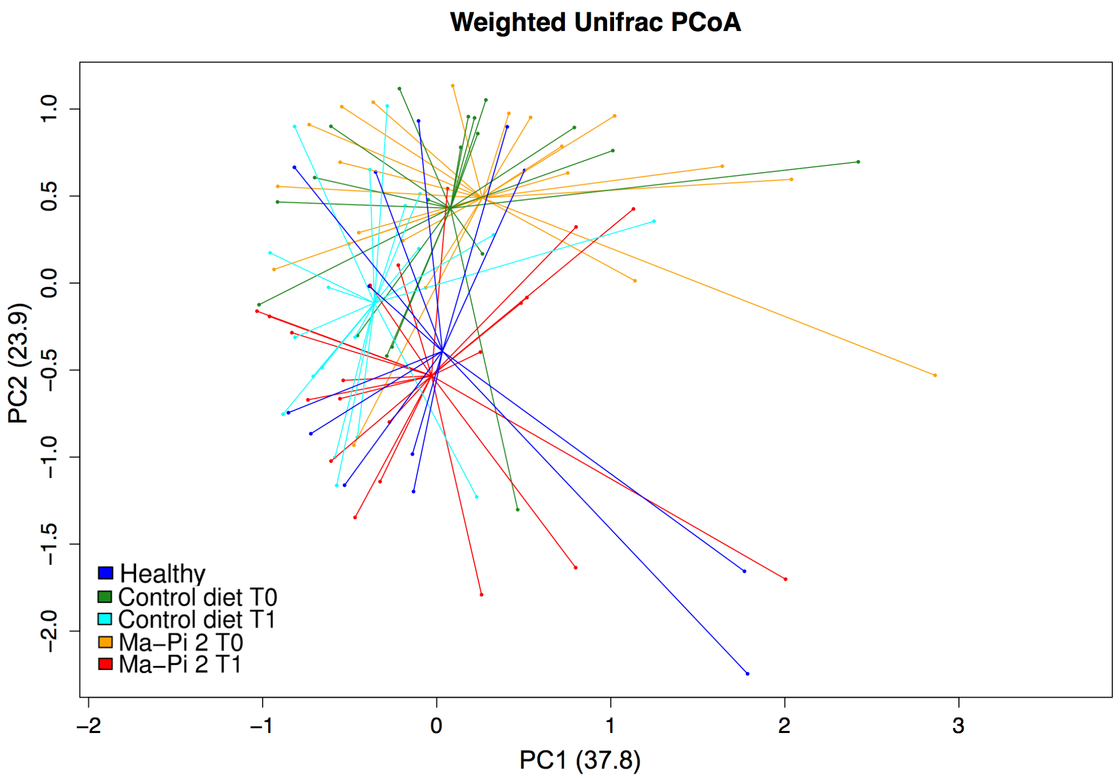

Supplemental Figure 3 : PCoA analysis of the weighted UniFrac distances between healthy subjects (blue), CTR diet subjects at T0 (green) and T1 (cyan), , Ma-Pi 2 subjects at T0 (orange) and T1 (red).
