# Supplementary material for: Modulation of gut microbiota dysbioses in type 2 diabetic patients by macrobiotic Ma-Pi 2 diet
Source: Br J Nutr. 2016 May 6;116(1):80–93. doi: 10.1017/S0007114516001045 (PMC4894062; doi:10.1017/S0007114516001045)
Supplement: Supplementary file 1 [file S0007114516001045sup.zip › S0007114516001045sup004.pdf]

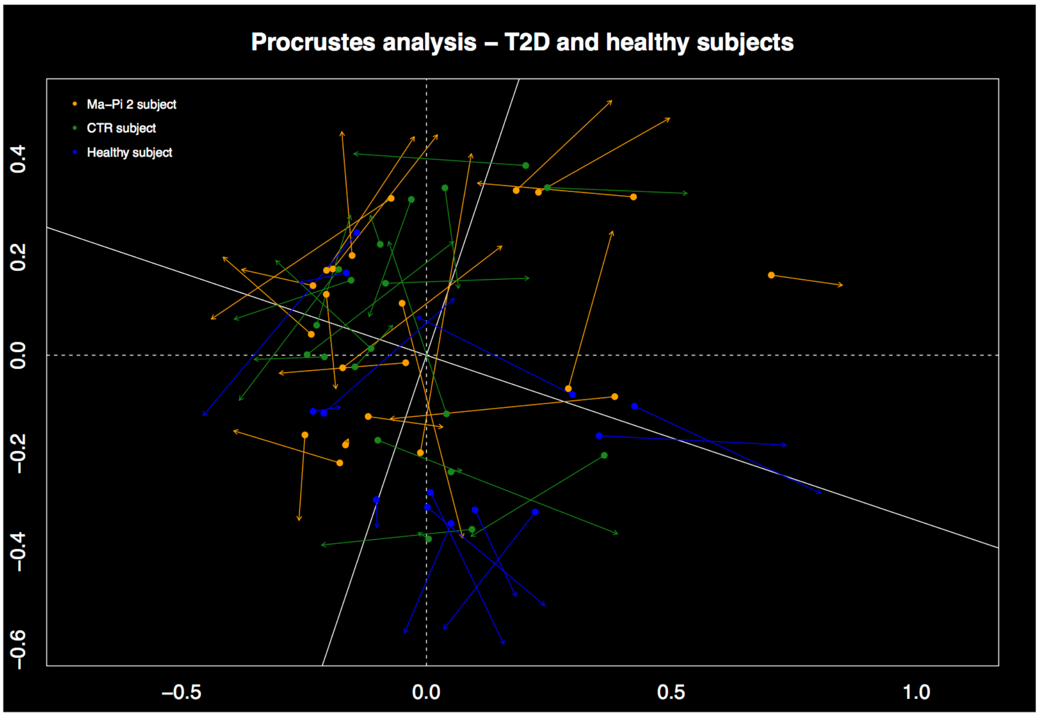

Supplemental Figure 4: procrustes analysis of the 16S rDNA sequences and imputed KO gene dataset supporting the significant association between taxonomic and inferred functional profiles of the gut microbiome across our cohorts (Protest significance = 0.001).
