# Supplementary material for: Modulation of gut microbiota dysbioses in type 2 diabetic patients by macrobiotic Ma-Pi 2 diet
Source: Br J Nutr. 2016 May 6;116(1):80–93. doi: 10.1017/S0007114516001045 (PMC4894062; doi:10.1017/S0007114516001045)
Supplement: Supplementary file 1 [file S0007114516001045sup.zip › S0007114516001045sup006.docx]

**Supplemental Table 1:** Nutritional intake across dietary habits and intervention diets. Daily intake of energy and macronutrients (fat, protein, carbohydrate and fiber) was calculated for each dietary regimen. Pre-intervention diet of T2D patients and the dietary habits of healthy controls were assessed using qualitative and quantitative food questionnaires.

| Dietary habits | Energy (kcal) | Fat  (%) | Protein (%) | Carbohydrate (%) | Fiber  (g/1000 kcal) |
| --- | --- | --- | --- | --- | --- |
| T2D patients (pre-intervention) | 1964-1988 | 35.4-36.2 | 18.2-19.3 | 45.3-45.6 | 10.3-10.8 |
| Healthy controls | 1852 | 11.3 | 12.9 | 75.7 | 10.7 |
| Intervention diets for T2D patients | Energy (kcal) | Fat  (%) | Protein (%) | Carbohydrate (%) | Fiber  (g/1000 kcal) |
| Ma-Pi 2 diet | 1803 | 15.2 | 11.8 | 73.0 | 29.0 |
| CTR diet | 1798 | 32.3 | 18.4 | 49.3 | 20.5 |
