# Supplementary material for: Modulation of gut microbiota dysbioses in type 2 diabetic patients by macrobiotic Ma-Pi 2 diet
Source: Br J Nutr. 2016 May 6;116(1):80–93. doi: 10.1017/S0007114516001045 (PMC4894062; doi:10.1017/S0007114516001045)
Supplement: Supplementary file 1 [file S0007114516001045sup.zip › S0007114516001045sup007.docx]

**Supplemental Table 2:** Food category distribution across nutritional interventions. Categories are reported as a percentage of a 2-d diet for Ma-Pi 2 and CTR diet. The daily caloric intake was 1700 kcal for women and 1900 kcal for men. Food categories are reported as in Schnorr et al. ^(60)^.

| **Food category** | **Ma-Pi 2 1900 kcal (g/2 d)** | **Ma-Pi 2 1700 kcal (g/2 d)** |
| --- | --- | --- |
| Bread, cereal, pasta, grains | 0.0% | 0.0% |
| Other starch | 2.8% | 3.2% |
| Whole grain | 27.2% | 29.0% |
| Meats, eggs, meat broths | 0.0% | 0.0% |
| Dairy | 0.0% | 0.0% |
| Fruit, fruit juice | 0.0% | 0.0% |
| Vegetable, sauces, herbs | 65.9% | 63.2% |
| Seeds | 2.6% | 2.9% |
| Seaweeds | 0.3% | 0.3% |
| Fermented products | 1.2% | 1.4% |
| Extra virgin olive oil | 0.0% | 0.0% |
| TOTAL % | 100.0% | 100.0% |
| TOTAL g | 2827.8 | 2376.8 |
|  |  |  |
| **Food category** | **CTR 1900 kcal (g/2 d)** | **CTR 1700 kcal (g/2 d)** |
| Bread, cereal, pasta, grains | 4.8% | 5.1% |
| Other starch | 5.5% | 5.5% |
| Whole grain | 9.2% | 7.9% |
| Meats, eggs, meat broths | 9.7% | 7.3% |
| Dairy | 12.0% | 12.7% |
| Fruit, fruit juice | 24.7% | 26.2% |
| Vegetable, sauces, herbs | 31.1% | 32.9% |
| Seeds | 0.0% | 0.0% |
| Seaweeds | 0.0% | 0.0% |
| Fermented products | 0.0% | 0.0% |
| Extra virgin olive oil | 3.0% | 2.4% |
| TOTAL % | 100.0% | 100.0% |
| TOTAL g | 3496.0 | 3301.0 |
