# Supplementary material for: Modulation of gut microbiota dysbioses in type 2 diabetic patients by macrobiotic Ma-Pi 2 diet
Source: Br J Nutr. 2016 May 6;116(1):80–93. doi: 10.1017/S0007114516001045 (PMC4894062; doi:10.1017/S0007114516001045)
Supplement: Supplementary file 1 [file S0007114516001045sup.zip › S0007114516001045sup008.docx]

**Supplemental Table 3:** List of KEGG pathways significantly clustering with T2D patients or healthy controls, and responding to the Ma-Pi 2 diet. See biplot analysis of Figure 6 and 7.

| **Comparison T2D patients at baseline / healthy controls** | |
| --- | --- |
| **Functions associated with T2D** | **Functions associated with healthy controls** |
| Alanine, aspartate and glutamate metabolism | D-arginine and D-ornithine metabolism |
| Alpha linolenic acid metabolism | D-glutamine and D-glutamate metabolism |
| Arachidonic acid metabolism | Fatty acid biosynthesis |
| Pantothenate and CoA biosynthesis | Glycolysis / Gluconeogenesis |
| Phenylpropanoid biosynthesis |  |
| Polyketide sugar unit biosynthesis |  |
| Purine metabolism |  |
| Tyrosine metabolism |  |
| **Comparison T2D patients before / after Ma-Pi 2 diet** | |
| **Functions associated with T1** | **Functions associated with T0** |
| Alanine, aspartate and glutamate metabolism | Arachidonic acid metabolism |
| Biosynthesis of unsaturated fatty acids | Beta alanine metabolism |
| Cysteine and methionine metabolism | Cyanoamino acid metabolism |
| D-glutamine and D-glutamate metabolism | D-alanine metabolism |
| Glycolysis / Gluconeogenesis | Glycosphingolipid biosynthesis |
| Purine metabolism | Histidine metabolism |
| Taurine and hypotaurine metabolism | Oxidative phosphorylation |
| Valine, leucine and isoleucine degradation | Polyketide sugar unit biosynthesis |
|  | Sphingolipid metabolism |
